# Stability in Ecosystem Functioning across a Climatic Threshold and Contrasting Forest Regimes

**DOI:** 10.1371/journal.pone.0016134

**Published:** 2011-01-18

**Authors:** Elizabeth S. Jeffers, Michael B. Bonsall, Kathy J. Willis

**Affiliations:** 1 School of Geography, University of Oxford, Oxford, United Kingdom; 2 Department of Zoology, University of Oxford, Oxford, United Kingdom; 3 Department of Biology, University of Bergen, Bergen, Norway; Umea University, Sweden

## Abstract

Classical ecological theory predicts that changes in the availability of essential resources such as nitrogen should lead to changes in plant community composition due to differences in species-specific nutrient requirements. What remains unknown, however, is the extent to which climate change will alter the relationship between plant communities and the nitrogen cycle. During intervals of climate change, do changes in nitrogen cycling lead to vegetation change or do changes in community composition alter the nitrogen dynamics? We used long-term ecological data to determine the role of nitrogen availability in changes of forest species composition under a rapidly changing climate during the early Holocene (16k to 8k cal. yrs. BP). A statistical computational analysis of ecological data spanning 8,000 years showed that secondary succession from a coniferous to deciduous forest occurred independently of changes in the nitrogen cycle. As oak replaced pine under a warming climate, nitrogen cycling rates increased. Interestingly, the mechanism by which the species interacted with nitrogen remained stable across this threshold change in climate and in the dominant tree species. This suggests that changes in tree population density over successional time scales are not driven by nitrogen availability. Thus, current models of forest succession that incorporate the effects of available nitrogen may be over-estimating tree population responses to changes in this resource, which may result in biased predictions of future forest dynamics under climate warming.

## Introduction

Classical ecological theory posits that changes in the availability of nitrogen, an essential resource, can lead to changes in the species composition of plant communities due to differences in species-specific nutrient requirements [1]. This understanding is well-supported by studies of short-lived plant species for which long-term studies provide data on multiple generations of plant populations (ex. grasslands [2]). Yet, our understanding of the responses of long-lived species such as trees to changes in nitrogen availability is based primarily on studies of the growth response of individual trees or seedlings and saplings to experimental changes in nitrogen availability [3], [4], [5] that are scaled-up to the population level [6]. Such studies suggest that tree population growth is strongly dependent on available nitrogen and that populations have strong self-reinforcing feedback effects on this resource via differences in plant litter chemistry [7]. However, it remains unclear whether growth responses by individual trees are a good predictor of expected population-scale patterns given changes in the availability of essential nutrients [8]. Given that future climate warming is expected to increase rates of nitrogen cycling [9], it is essential to assess the effect of climate-induced changes in nitrogen cycling on changes on tree population dynamics [10] over time scales appropriate for long-lived plant species (i.e. 100's to 1,000's of years) [11]. Here, we use a mechanistic modelling analysis of palaeoecological data to determine if – during an interval of rapid climate change – changes in nitrogen dynamics lead to vegetation change or if community composition alters nitrogen dynamics.

Long-term ecological data from the fossil record yields reliable proxies of plant population dynamics [12] and changes in landscape-scale nitrogen cycling [13]. Recent palaeoecological studies have shown a high level of concurrence between changes in the nitrogen cycle and shifts in vegetation composition [14]. When these changes occur concurrently it is not possible to assess the direction of causation; therefore, it is essential to analyze the mechanistic relationships underlying the changes in vegetation composition and nitrogen cycling. We aimed to infer this mechanistic relationship and to test the stability of it over a threshold change in climate and a change in the dominant tree species on the landscape using a model-fitting and model-selection analysis of long-term ecological data.

We used palaeoecological data spanning 8,000 years from Kis-Mohos Tó in Northeast Hungary [15] that was interrupted by a threshold change in climate involving abrupt warming of 10°C over a 60 year period [16]. We tested the goodness of fit of three sets of candidate mechanistic models for explaining the dynamics of two dominant tree populations, *Pinus spp.* (pine) and *Quercus spp.* (oak), with changes in the nitrogen cycle: nitrogen-dependent population growth, nitrogen-dependent population growth with a positive feedback effect and nitrogen independent population growth with a positive feedback effect (see Table S1 for equations). We determined the relative strength of each mechanistic model in terms of its goodness of fit to the data by using an information criterion [17]; this form of analysis indicates the most likely mechanistic relationship between each tree population and the nitrogen cycle. Furthermore, the tested the effect of a nonlinear change in the trajectory of climate dynamics on plant-nitrogen cycle interactions.

## Results

### Palaeoecological Reconstruction

**Figure 1:**
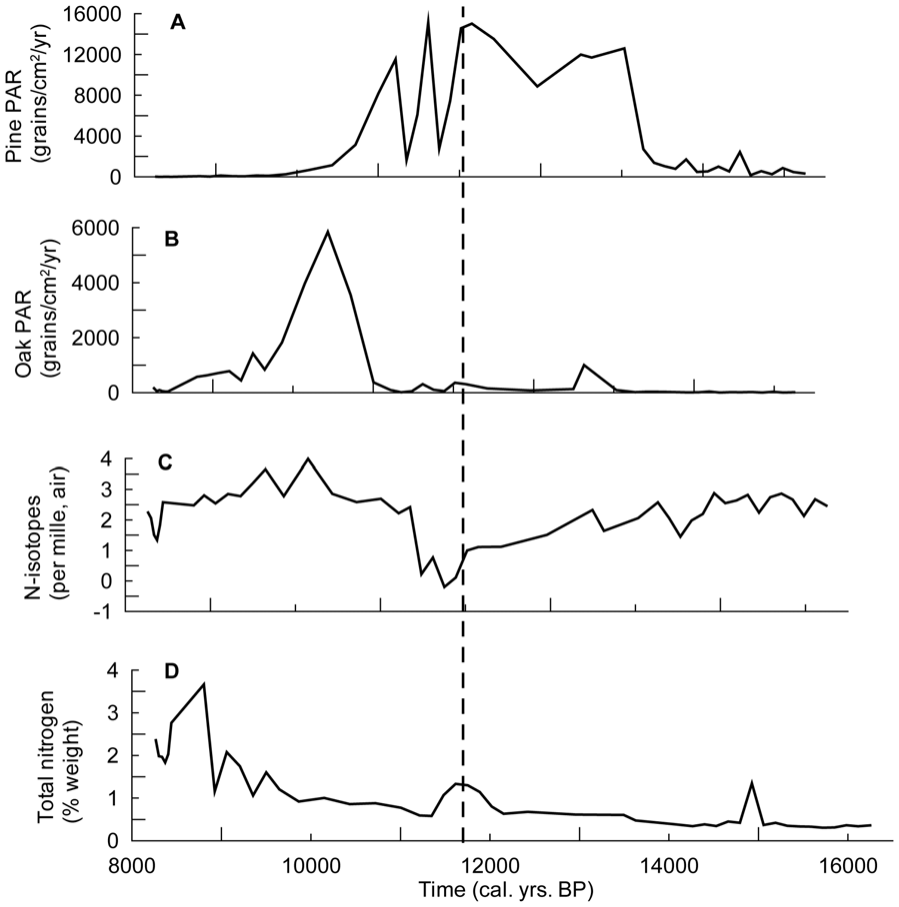
Ecosystem dynamics. Tree population dynamics are represented by pollen accumulation rates (PAR) of pine (A) and oak (B). Nitrogen dynamics reflect changes in nitrogen availability inferred from stable nitrogen isotope analysis (C) and changes in total nitrogen (D) inferred from elemental analysis of bulk organic matter. Abrupt warming occurred at 11.7k cal. yrs. BP (dotted line).

Tree population changes were reconstructed with fossil pollen accumulation rates (PAR) from lake sediments; PAR is a proven indicator of past changes in tree species density [18]. Pine was present in the landscape from 16,000 cal. yr. BP and became dominant between 14,000 and 12,000 cal. yr. BP (see Figure 1.A). Oak was also present in the landscape in low abundance from 16,000 cal. yr. BP and it had a short-lived period of expansion around 14,000 cal. yr. BP. Oak became dominant on the landscape at around 11,000 cal. yr. BP. This represented an ecosystem shift from coniferous-dominated to deciduous-dominated forest following rapid climate warming at 11,700 cal. yr. BP [16].

Changes in the nitrogen cycle were reconstructed by two different proxies: stable nitrogen isotopes (δ^15^N) and elemental concentrations of total nitrogen (%TN) within the sediments. Stable nitrogen isotopes have been shown to correspond with changes in available nitrogen in the terrestrial ecosystem [13]. Total nitrogen primarily represents the amount of nitrogen being transported into the lake from the surrounding catchment [19], and includes both plant-available and plant-unavailable forms of nitrogen. Coincident with the abrupt change in climate, there was a drop in δ^15^N-inferred nitrogen availability (Figure 1.C) that was coincident with a temporary increase in total nitrogen (%TN, Figure 1.D). This suggests that a short-term increase in the amount of nitrogen entering the lake occurred with the rapid climate change. By 11,500 cal. yr. BP, when %TN reduced to previous levels there was a corresponding increase in δ^15^N, which exceeded its pre-12,000 cal. yr. BP level. By this time, oak had become the dominant tree species in the forest and the pine population began a rapid decline. During the time of oak-dominance, δ^15^N remained high and %TN increased gradually, yet at a faster rate than under pine-dominance. Oak dominance lasted until after 10,000 cal. yr. BP, when other broadleaved trees such as hazel, lime and elm dominated the ecosystem [15]; this transition corresponded with rapid and significant increases in %TN and continued high values of δ^15^N, which suggest that high amounts of nitrogen were entering the lake system.

### Mechanistic Relationships

We used Akaike Information Criterion (AIC) weights [17] to demonstrate the relative amount of support for each mechanistic model of tree population-nitrogen cycle dynamics. Furthermore, to establish whether or not there is evidence of a threshold change in ecosystem functioning coincident with the change in climate at 11,700 cal. yrs. BP, we further separated the coupled nitrogen-tree population models into two types: a threshold model, where the models were fitted separately to the pre- and post- climatic threshold periods; and a non-threshold model where ecosystem functioning was assumed to be constant over the entire series.

The AIC-best model for describing the relationship between both the pine and the oak populations and the nitrogen cycle was the nitrogen independent population growth with feedback effects (i.e. plant-driven nitrogen cycle) model, where the interaction between the population dynamics and the nitrogen cycle occurs via declining plant litter (Table 1). This was the most-likely mechanism by which the populations interacted with the nitrogen cycle in both the threshold and non-threshold cases. Therefore, this form of interaction between the dominant vegetation and the nitrogen cycle held true despite a threshold in climate being passed and a shift from coniferous-dominated ecosystem to a deciduous-dominated one.

**Table 1:** Most likely relationship between each plant population and each nitrogen proxy based on Akaike weights (*w_i_*), where each column sums to 100.

	Non-threshold Model				Threshold Model			
	*w_i_* Pine		*w_i_* Oak		*w_i_* Pine		*w_i_* Oak	
	δ^15^N	%TN	δ^15^N	%TN	δ^15^N	%TN	δ^15^N	%TN
Nitrogen-dependent population growth	0.00%	0.00%	0.02%	0.00%	0.16%	0.01%	0.11%	19.42%
Nitrogen-dependent population growth with feedback effect	0.00%	0.00%	0.03%	0.00%	1.79%	0.01%	2.85%	23.42%
Nitrogen independent population growth with feedback effect	100%	100%	99.95%	100%	98.05%	99.97%	97.05%	57.15%

The ability of the threshold and non-threshold versions of the models to predict the observed relationships between the tree population dynamics and the nitrogen cycle was determined by calculating and comparing the relative AIC weights of the threshold versus non-threshold versions of the plant-nitrogen cycle models. The threshold version of the model provided a better fit to the data than the non-threshold version: AIC weights indicate that threshold models have 100% of support from the data and this held true for both the oak and pine populations.

### Changes in Plant-Nitrogen Relationship

**Figure 2:**
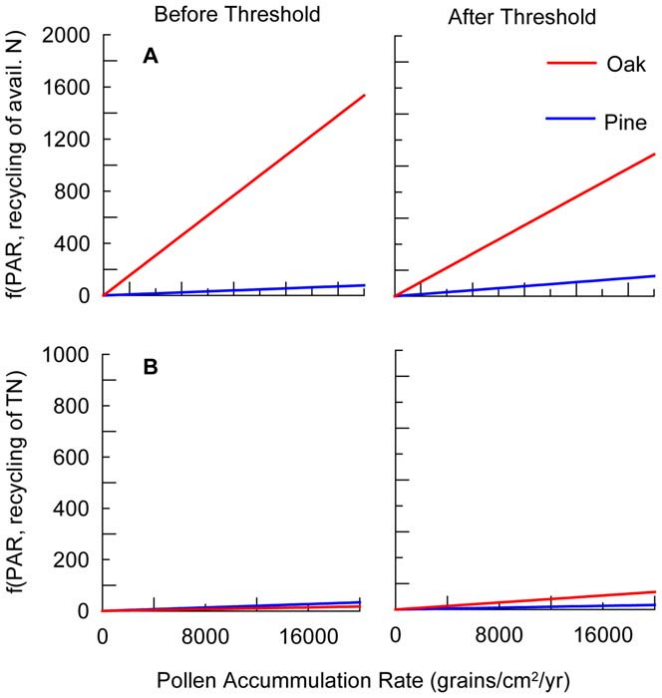
Ecosystem engineering effects of oak and pine. Effect of changes in biomass (inferred from pollen accumulation rates (PAR))on available nitrogen (A) and total nitrogen (B) given maximum likelihood estimates of species-specific mortality rates and rates of conversion of biomass loss into greater amounts of the resource.

Following the threshold change in climate at 11,700 cal. yrs. BP, there were notable changes in the estimated parameters for both tree populations (Table 2). Maximum likelihood estimated parameters of the AIC-inferred best plant-nitrogen cycle model suggest that climate warming was associated with increased rates of conversion of decaying biomass of both species into available nitrogen, increased background loss rates of available nitrogen but decreased background loss rates of total nitrogen. There was no change in the rate of conversion of decaying biomass of pine on total nitrogen following warming while there was an increase in the rate of conversion of decaying oak biomass into total nitrogen. There were increases in the population growth rates of both species with warming after 11.7k cal. yrs. BP, but contrasting effects on loss rates: loss rates of oak decreased (although these parameters were not found to be significant) while loss rates of pine increased relative to the cool period prior to 11.7k cal. yrs. BP. The net effect of changing biomass on nitrogen cycling, as inferred by the product of the loss rates of each species and the conversion rates of their decaying biomass into available and total nitrogen show that the oak population had a consistently greater positive effect on both proxies of the nitrogen cycle than the pine population (Figure 2).

**Table 2:** Key parameters (and standard errors) of the plant-driven nitrogen cycle models for each tree population and each proxy of the nitrogen cycle for the periods before and after climate warming that occurred at 11,700 cal. yrs. BP.

	Pine and δ^15^N		Pine and %TN		Oak and δ^15^N		Oak and %TN	
Key Parameters	Pre-threshold	Post-threshold	Pre-threshold	Post-threshold	Pre-threshold	Post-threshold	Pre-threshold	Post-threshold
Growth rate of tree population (r_y_)	1510.215 (229.386)	2032.338 (313.156)	274.566 (31.855)	322.331(57.47)	177.375 (18.10)	82.517 (102.007)	98.78 (8.313)	272.064 (89.345)
Loss rate of tree population (γ_y_)	3.371 (1.206)	4.181 (1.697)	0.00088 (0.0005)	0.001 (0.0002)	74.475 (5.515)	11.453 (39.8)	28.933 (7.844)	5.522 (41.533)
Conversion of decaying tree biomass into soil nitrogen (α)	0.001 (0.000252)	0.002 (0.000398)	0.002 (0.0006)	0.002 (0.00024)	0.001 (0.00011)	0.005 (0.004)	0.00087 (0.00017)	0.003 (0.002)
Loss rate of nitrogen from landscape (γ_x_)	12.388 (0.952)	14.07 (0.342)	8.178 (2.918)	3.464 (0.065)	3.954 (0.599)	23.595 (19.75)	5.75 (0.11)	13.035 (9.024)

## Discussion

Contrary to current thinking, results from our study suggest that the nitrogen cycle was not a driver of secondary succession in this ecosystem during the period of rapid and significant climatic warming at the late-glacial/early postglacial transition. The threshold change in climate was associated with a decrease in the predicted mortality rates of long-lived oak trees, resulting in reduced opportunities for replacement by pine [20]. Given *Quercus spp.*'s greater shade tolerance than *Pinus spp.* [21] we suggest that competition for light may have been the process by which oak replaced pine following the threshold change in warming. Our finding that secondary succession occurred independently of changes in nitrogen availability supports the findings of Menge et al. [22] that forest ecosystems are not limited by available nitrogen over long time scales. These results however are in contrast with the assumptions made in select dynamic global vegetation models (DGVMs) that available nitrogen can limit tree population growth [23] during climate warming.

Our results demonstrated stability in ecosystem functioning across the interval of rapid climatic change and despite a change in the dominant tree species. Climate change was found to affect the rates of nitrogen cycling: following the threshold change in climate, our model indicated that there were higher rates of conversion of decaying biomass into available and total nitrogen. The effect of changes in biomass on the nitrogen cycle was modified by concurrent changes in the loss rates of both species; yet, there was a consistently greater effect of increasing oak density on available and total nitrogen, which concurs with contemporary studies of nitrogen concentrations in leaves/needles in *Quercus spp.* and *Pinus spp.* [24] and differences in nitrogen resorption proficiency [25]. Thus, while the mechanism underlying both species' interaction with the nitrogen cycle remained constant, the observed vegetation changes led to increased rates of nitrogen cycling given warming and the shift to oak-dominated landscape even though this feedback effect was not found to have a demonstrable impact on the fitness of either population at the millennial time scale.

It is important for future work to test the context-dependence of our findings by validation with other appropriate palaeoecological datasets of coniferous to deciduous forest transitions and changes in nitrogen cycling during the late-glacial warming period. Furthermore, in order for model-fitting and model-selection analysis of palaeoecological data to yield sufficient information to be useful for predictive modelling, this approach needs to be used to analyze a greater variety of transitions in dominant plant functional types. These caveats notwithstanding, results from this study demonstrate how the dynamics associated with ecosystem functioning can remain relatively stable following a major environmental perturbation.

## Materials and Methods

### Palaeoecological Analyses

The fossil pollen and stable isotope time series were derived from an early postglacial sediment sequence taken from a peat bog, Kis-Mohos Tó (20°24′30″E; 48°24′40″N), in Northeast Hungary [15]. The bottom 2.4 m of an undisturbed 8.86-m sedimentary sequence was collected from Kis-Mohos To′ and analyzed for a range of proxies, including fossil pollen, elemental concentrations and stable isotopes of nitrogen.

Seven radiocarbon dates for this section of the core were obtained by a combination of bulk and AMS (Accelerator Mass Spectrometry) analyses (see Table S2). These dates were calibrated using BCal [26] and the 2004 Northern Hemisphere ^14^C atmospheric calibration curve [27] and interpolated using linear interpolation.

Our time-series (n = 47) included measurements of all proxies (i.e. fossil pollen, elements and staple isotopes) taken from the same time periods. Samples were analyzed at a resolution of two to eight centimeters throughout the 2.4 m sediment core section. The sediment was prepared for pollen analysis following Berglund and Ralska-Jasiewiczowa [28]; this method involves adding a known quantity of exotic pollen, which allows for the determination of pollen concentration [29]. In order for our pollen data to provide the most appropriate representation of past tree population densities, we converted the pollen concentration values into pollen accumulation rates (PAR) [18], [30], [31]: the pollen concentration (grains/cm^3^) divided by the sediment accumulation rate (cm^2^/yr) giving PAR (grains/cm^2^/yr).

Elemental (%TN) and stable nitrogen isotope (δ^15^N) analysis followed the methods described in Talbot [19]. Each bulk sample was dried at <40°C and then ground with a pestle and mortar. About 40 mg of each dried and homogenized sample was transferred to a small plastic vial and sent to the Research Laboratories for Ancient History and Archeology (RLAHA) at the University of Oxford where they were analyzed by mass spectrometry.

### Non-Linear Population Dynamic Models

#### Candidate Models

There were three groups of candidate models used in this study: nitrogen-dependent population growth, nitrogen-dependent population growth with a positive feedback effect of decaying plant biomass on nitrogen cycling and nitrogen independent population growth with the same feedback effect. These different groups of models were used to test the hypothesis of whether or not the populations were dependent on nitrogen for population growth and whether or not they had a feedback effect on the nitrogen cycle.

Within each group we applied different versions of the model to the data in order to detect the best functional form for describing the relationship between the populations and the nitrogen cycle (for the nitrogen-dependent population growth models). In particular, we applied two different plant-resource uptake models to determine which of these provided the best explanation of the joint plant population-nitrogen cycle dynamics: saturating uptake and linear uptake (see Table S1).

To test whether or not there was evidence that density-dependent population growth limited the population growth rates, we incorporated the logistic density dependence function in the population growth model and compared the goodness of fit of this model to the fit of the models without this function. In the case of the nitrogen independent population growth models, we varied the functional form of the density-dependent control on population growth between a logistic and an exponential function.

#### Model-Fitting

The model-fitting approach uses maximum likelihood estimation to find the set of parameters that yield the best fit to the nitrogen and tree species' dynamics data. Model-fitting was achieved by integrating a set of differential equations using a Runge-Kutta 4 numerical integration routine to generate expected values of the nitrogen availability and tree population data over variable time steps (*variable* due to the fact that there are not uniform time differences between observations in palaeoecological data). The model was initialized with the first measurement from the data, and expected values were generated from user-defined initial parameter values. For example, using the model of saturating resource-dependence without a density-dependent effect on growth, the following set of equations would be integrated:
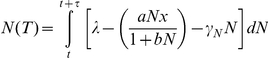


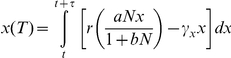
to give the expected values of nitrogen (*N(T)*) and vegetation (*x(T)*) at each census point (*t*). *τ* represents the variable integration time-step between census points, which is equivalent to the time lapsed between observations in the palaeoecological record. The expected values change as the set of model parameters, **P** (e.g. **P** = [*λ, a, b, γ_N_, r, γ_x_*] for the above model), are varied.

A maximum likelihood method is used to determine the set of model parameters, **P**, that provide the best fit of each model to the data (i.e. that minimizes the difference between the model-generated data and the observed data). The following likelihood function is used to evaluate the goodness-of-fit of each model:



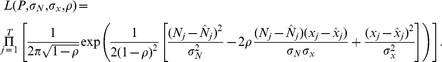



This likelihood function assumes that model errors are normally distributed and that stochastic events on each population are primarily driven by external environmental effects [32]. Evaluating the likelihood function involves estimating the set of model parameters (**P**), the standard deviation associated with nitrogen abundance (*σ_N_*), the standard deviation associated with tree abundance (*σ_x_*) and their covariance (*ρ*). At each time step *j* the expected values (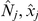) are compared to the observed values 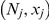 with the objective of finding the set of model parameters, **P**, that minimize the difference between these observed and expected values. This was accomplished by using a simplex optimization routine implemented in C [32], which searched for the set of **P** that minimized the negative log-likelihood value. Confidence intervals for the maximum likelihood estimated (MLE) parameters (**P**) were calculated from the likelihood profiles following Morgan [33].

To determine if there was a threshold change in the relationship between tree population density and the nitrogen cycle, a threshold was simulated by splitting the data at the point where rapid climatic warming is known to have occurred (i.e., around 11,700 cal. yr. BP). The ten models (Table S1) were fitted separately to each segment of the split (i.e. threshold) dataset (n_1_ = 24, n_2_ = 23), which allowed the parameters to vary across this period of climatic change. The likelihood of the threshold models was determined by summing the likelihoods of each segment. The same models were also applied to the whole dataset (n_whole_ = 47), where the parameters were held constant over the entire series.

#### Model Selection

Model-selection was used to determine the model of ecosystem functioning that provided the best representation of the tree population density and nitrogen cycling data. The model-fitting step provided a negative log-likelihood value for each model; this was used to calculate an Information Criterion score for each model. The Akaike Information Criterion (AIC) was used, which is a weighted goodness of fit criterion [17]. The AIC score was converted into a normalized indicator of support for each model by calculating AIC weights (*w_i_*), where larger values indicate greater evidence in support of the model.

## Supporting Information

Table S1 Sets of differential equations used to describe tree population and nitrogen dynamics.
(DOC)

Table S2 Radiocarbon dates.
(DOC)

## References

[1] Tilman D, May RM, McLean AR (2007). Interspecific competition and multispecies coexistence.. Theoretical Ecology: principles and applications. 3rd ed.

[2] Reich PB, Tilman D, Craine J, Ellsworth D, Tjoelker MG (2001). Do species and functional groups differ in acquisition and use of C, N and water under varying atmospheric CO2 and N availability regimes? A field test with 16 grassland species.. New Phytologist.

[3] Wallace ZP, Lovett GM, Hart JE, Machona B (2007). Effects of nitrogen saturation on tree growth and death in a mixed-oak forest.. Forest Ecology and Management.

[4] Magill AH, Aber JD, Hendricks JJ, Bowden RD, Melillo JM (1997). Biogeochemical response of forest ecosystems to simulated chronic nitrogen deposition.. Ecological Applications.

[5] Catovsky S, Bazzaz FA (2002). Nitrogen availability influences regeneration of temperate tree species in the understory seedling bank.. Ecological Applications.

[6] Catovsky S, Kobe RK, Bazzaz FA (2002). Nitrogen-induced changes in seedling regeneration and dynamics of mixed conifer-broad-leaved forests.. Ecological Applications.

[7] Clark BR, Hartley SE, Suding KN, de Mazancourt C (2005). The effect of recycling on plant competitive hierarchies.. American Naturalist.

[8] Suding KN, Lavorel S, Chapin FS, Cornelissen JHC, Diaz S (2008). Scaling environmental change through the community-level: a trait-based response-and-effect framework for plants.. Global Change Biology.

[9] Gruber N, Galloway JN (2008). An Earth-system perspective of the global nitrogen cycle.. Nature.

[10] Smith MD, Knapp AK, Collins SL (2009). A framework for assessing ecosystem dynamics in response to chronic resource alterations induced by global change.. Ecology.

[11] Bardgett RD, Bowman WD, Kaufmann R, Schmidt SK (2005). A temporal approach to linking aboveground and belowground ecology.. Trends in Ecology & Evolution.

[12] Green DG (1981). Time-series and post-glacial forest ecology.. Quaternary Research.

[13] McLauchlan KK, Craine JM, Oswald WW, Leavitt PR, Likens GE (2007). Changes in nitrogen cycling during the past century in a northern hardwood forest.. Proceedings of the National Academy of Sciences of the United States of America.

[14] Wolfe BB, Edwards TWD, Aravena R (1999). Changes in carbon and nitrogen cycling during tree-line retreat recorded in the isotopic content of lacustrine organic matter, western Taimyr Peninsula, Russia.. Holocene.

[15] Willis KJ, Braun M, Sumegi P, Toth A (1997). Does soil change cause vegetation change or vice versa? A temporal perspective from Hungary.. Ecology.

[16] Steffensen JP, Andersen KK, Bigler M, Clausen HB, Dahl-Jensen D (2008). High-resolution Greenland Ice Core data show abrupt climate change happens in few years.. Science.

[17] Burnham KP, Anderson DR (2002). Model selection and multimodel inference: a practical information-theoretic approach..

[18] Prentice IC (1988). Paleoecology and Plant-Population Dynamics.. Trends in Ecology & Evolution.

[19] Talbot MR, Last WM, Smol JP (2001). Nitrogen istopes in palaeolimnology.. Tracking Environmental Change Using Lake Sediments; Vol 2.

[20] Connell JH, Slatyer RO (1977). Mechanisms of Succession in Natural Communities and Their Role in Community Stability and Organization.. American Naturalist.

[21] Niinemets U, Valladares F (2006). Tolerance to shade, drought, and waterlogging of temperate Northern Hemisphere trees and shrubs.. Ecological Monographs.

[22] Menge DNL, Pacala SW, Hedin LO (2009). Emergence and Maintenance of Nutrient Limitation over Multiple Timescales in Terrestrial Ecosystems.. American Naturalist.

[23] Xu R, Prentice IC (2008). Terrestrial nitrogen cycle simulation with a dynamic global vegetation model.. Global Change Biology.

[24] Karolewski P, Giertych MJ, Oleksyn J, Zytkowiak R (2005). Differential reaction of Pinus sylvestris, Quercus robur and Q-petraea trees to nitrogen and sulfur pollution.. Water Air and Soil Pollution.

[25] Eckstein RL, Karlsson PS, Weih M (1999). Leaf life span and nutrient resorption as determinants of plant nutrient conservation in temperate-arctic regions.. New Phytologist.

[26] Buck CE, Christen JA, James GN (1999). BCal: an on-line Bayesian radiocarbon calibration tool.. Internet Archaeology.

[27] Reimer PJ, Baillie MGL, Bard E, Bayliss A, Beck WJ (2004). IntCal04 Terrestrical Radiocarbon Age Calibration, 0-26 cal kyr BP.. Radiocarbon.

[28] Berglund BE, Ralska-Jasiewiczowa M, International Geological Correlation Programme. Project 158B (1986). Handbook of Holocene palaeoecology and palaeohydrology..

[29] Bennett KD, Willis KJ, Smol JP, Birks HJB, Last WM (2001). Pollen.. Tracking Environmental Change Using Lake Sediments.

[30] Delcourt HR, Delcourt PA (1991). Quaternary ecology: a paleoecological perspective..

[31] Green DG (1983). The ecological interpretation of fine resolution pollen records.. New Phytologist.

[32] Bonsall MB, Hastings A (2004). Demographic and environmental stochasticity in predator-prey metapopulation dynamics.. Journal of Animal Ecology.

[33] Morgan BJT (1999). Applied stochastic modelling..

