# Supplementary material for: Stability in Ecosystem Functioning across a Climatic Threshold and Contrasting Forest Regimes
Source: PLoS One. 2011 Jan 18;6(1):e16134. doi: 10.1371/journal.pone.0016134 (PMC3022756; doi:10.1371/journal.pone.0016134)
Supplement: Table S1 — Sets of differential equations used to describe tree population and nitrogen dynamics. (DOC) [file pone.0016134.s001.doc]

**Table S1.** Sets of differential equations used to describe tree population and nitrogen dynamics

| MODEL | EQUATIONS |
| --- | --- |
| *Nitrogen Uptake*  Saturating Uptake      Linear Uptake    Saturating Uptake + Density Dependent Growth    Linear Uptake + Density Dependent Growth | $\frac{dN}{dt}=\lambda-\frac{aNx}{1+bN}-\gamma_{N}N$;  $\frac{dx}{dt}=r\frac{aNx}{1+bN}-\gamma_{x}x$ $\frac{dN}{dt}=\lambda-aNx-\gamma_{N}N$;  $\frac{dx}{dt}=raNx-\gamma_{x}x$ $\frac{dN}{dt}=\lambda-\frac{aNx}{1+bN}-\gamma_{N}N$;  $\frac{dx}{dt}=r\frac{aNx}{1+bN}(1-\frac{x}{K})-\gamma_{x}x$ $\frac{dN}{dt}=\lambda-aNx-\gamma_{N}N$; $\frac{dx}{dt}=raNx(1-\frac{x}{K})-\gamma_{x}x$ |
| *Nitrogen Uptake with Feedback*  Saturating Uptake      Linear Uptake    Saturating Uptake + Density Dependent Growth    Linear Uptake + Density Dependent Growth | $\frac{dN}{dt}=\lambda-\frac{aNx}{1+bN}-\gamma_{N}N+\alpha\gamma_{x}x$;  $\frac{dx}{dt}=r\frac{aNx}{1+bN}-\gamma_{x}x$ $\frac{dN}{dt}=\lambda-aNx-\gamma_{N}N+\alpha\gamma_{x}x$;  $\frac{dx}{dt}=raNx-\gamma_{x}x$ $\frac{dN}{dt}=\lambda-\frac{aNx}{1+bN}-\gamma_{N}N+\alpha\gamma_{x}x$;  $\frac{dx}{dt}=r\frac{aNx}{1+bN}(1-\frac{x}{K})-\gamma_{x}x$ $\frac{dN}{dt}=\lambda-aNx-\gamma_{N}N+\alpha\gamma_{x}x$; $\frac{dx}{dt}=raNx(1-\frac{x}{K})-\gamma_{x}x$ |
| *Plant-driven Nitrogen Cycle*  Logistic Density Dependence    Exponential Density Dependence | $\frac{dN}{dt}=\lambda-\gamma_{N}N+\alpha\gamma_{x}x$;  $\frac{dx}{dt}=rx(1-\frac{x}{K})-\gamma_{x}x$ $\frac{dN}{dt}=\lambda-\gamma_{N}N+\alpha\gamma_{x}x$; $\frac{dx}{dt}=rx*\exp(-ax)-\gamma_{x}x$ |
