# Supplementary material for: Stability in Ecosystem Functioning across a Climatic Threshold and Contrasting Forest Regimes
Source: PLoS One. 2011 Jan 18;6(1):e16134. doi: 10.1371/journal.pone.0016134 (PMC3022756; doi:10.1371/journal.pone.0016134)
Supplement: Table S2 — Radiocarbon dates. (DOC) [file pone.0016134.s002.doc]

**Table S2.** Radiocarbon dates

| Laboratory No. | Depth (cm) | Mean Depth  (cm) | Age (^14^C yr BP) | Age (cal. ^14^C yr BP) |
| --- | --- | --- | --- | --- |
| deb-3296  deb-3324  AA-11986  OxA-15342  OxA-15434  Poz-13161  Poz-13153 | 600 – 616  658 – 674  675  685  695  740  822 | 608  666  675  685  695  740  822 | 7,379 ± 82  7,685 ± 51  8,020 ± 100  8,750 ± 45  9,665 ± 45  12,310 ± 60  13,190 ± 70 | 8,189 ± 95  8,442 ± 47  8,987 ±152  9,721 ± 103  11,148 ± 125  14,166 ± 171  15,520 ± 183 |
